# Imlunestrant with or without abemaciclib in advanced breast cancer: safety analyses from the EMBER-3 trial

**DOI:** 10.1038/s41523-026-00950-z

**Published:** 2026-04-27

**Authors:** Joyce O’Shaughnessy, Francois-Clement Bidard, Patrick Neven, Monica Lis Casalnuovo, Philippe Aftimos, Cristina Saura, Nadia Harbeck, Lisa A. Carey, Giuseppe Curigliano, Jose A. Garcia-Saenz, Maria Fernandez Abad, Larissa de Paula, Yeon Hee Park, Ozgur Ozyilkan, Maria Munoz, Emily Barrett, Shanshan Cao, Aarti Chawla, Komal L. Jhaveri

**Affiliations:** 1 Baylor University Medical Center, Texas Oncology, Sarah Cannon Research Institute, Dallas, TX USA; 2 Institut Curie, Paris and Saint Cloud, Saint Cloud, France; 3 Department of Oncology, Universitaire Ziekenhuizen Leuven, Leuven, Belgium; 4 Hospital María Curie, Buenos Aires, Argentina; 5 Institut Jules Bordet, Hôpital Universitaire de Bruxelles (HUB), Brussels, Belgium; 6 Vall d’Hebron University Hospital, Vall d’Hebron Institute of Oncology (VHIO), Barcelona, Spain; 7 Breast Center, Department of Obstetrics and Gynecology and CCC Munich, LMU University Hospital, Munich, Germany; 8 University of North Carolina at Chapel Hill, Chapel Hill, NC USA; 9 Department of Oncology and Hemato-Oncology, University of Milano, Milano, Italy; 10 European Institute of Oncology, IRCCS, Milano, Italy; 11 Hospital Clinico Universitario San Carlos, Madrid, Spain; 12 Hospital Universitario Ramón y Cajal, Madrid, Spain; 13 Núcleo de Pesquisa do Instituto Brasileiro de Controle do Câncer (IBCC Oncologia), São Paulo, Brazil; 14 Samsung Medical Center, Sungkyunkwan University School of Medicine, Seoul, Republic of Korea; 15 Baskent University, Adana, Turkey; 16 Eli Lilly and Company, Indianapolis, IN USA; 17 Memorial Sloan Kettering Cancer Center, New York, NY USA; 18 Weill Cornell Medical College, New York, NY USA

**Keywords:** Cancer, Endocrinology, Oncology

## Abstract

In EMBER-3 (NCT04975308), among patients with ER + , HER2- advanced breast cancer (ABC) with recurrence/progression on/after endocrine therapy (ET), imlunestrant significantly prolonged PFS versus standard ET in patients with *ESR1* mutations, and imlunestrant + abemaciclib prolonged PFS versus imlunestrant in the overall population. We report the incidence, severity, timing, and management of common treatment-emergent adverse events (TEAEs), including safety profiles of select subgroups. In both imlunestrant-containing arms, the most common TEAEs were reversible, low grade, single occurrences; occurred early in treatment; and resulted in few treatment discontinuations (imlunestrant, 5%; imlunestrant + abemaciclib, 6%). TEAEs in imlunestrant+abemaciclib arm were managed with dose adjustments (61%) and supportive medication. Imlunestrant monotherapy had a similar safety profile between patients aged <65 and ≥65 years, while imlunestrant + abemaciclib had a similar safety profile to other abemaciclib+ET combinations in both age groups. Imlunestrant and imlunestrant + abemaciclib provide effective, convenient oral therapy with a favorable and manageable safety profile for patients with ER+, HER2- ABC with recurrence/progression on/after ET.

## Introduction

Endocrine therapy (ET) with or without a cyclin-dependent kinase 4/6 inhibitor (CDK4/6i) is the standard of care (SOC) for patients with estrogen receptor–positive (ER+), human epidermal growth factor receptor 2-negative (HER2-) advanced breast cancer (ABC)^1^. However, tumors eventually develop resistance to ET, causing metastatic relapse and disease progression. Selective ER degraders (SERDs), a class of ET, induce the degradation of ER alpha, a key oncogenic driver of estrogen signaling in breast cancer^2–4^.

Although fulvestrant, the first approved SERD^5–8^, is generally well tolerated, its clinical utility is limited by its pharmacokinetic properties and intramuscular administration, which in turn affects its dosage^9,10^. Next-generation oral SERDs are currently in development, with elacestrant being the first approved. While these agents have demonstrated improved efficacy, particularly in tumors with an *ESR1* mutation (*ESR1*m)—an acquired mutation in up to 50% of patients who have received prior aromatase inhibitor (AI) therapy^11–13^—the use of some therapeutics within this class has been associated with unique toxicities such as bradycardia, QT interval prolongation, and visual disturbances^12,14^.

Imlunestrant is a next-generation, orally bioavailable, brain-penetrant SERD with pure antagonistic properties resulting in sustained inhibition of ER-dependent gene transcription and cell growth^15,16^. At the primary analysis of the phase III EMBER-3 trial in patients with ER+, HER2- ABC with disease recurrence or progression during or after AI therapy alone or with a CDK4/6i, imlunestrant significantly improved progression-free survival (PFS) versus SOC ET (fulvestrant or exemestane) in patients with *ESR1*m tumors (5.5 vs 3.8 months; hazard ratio [HR]: 0.62; 95% confidence interval [CI]: 0.46–0.82; *P* < 0.001)^17^. The combination of imlunestrant + abemaciclib significantly improved PFS versus imlunestrant alone in all patients (9.4 vs 5.5 months; HR: 0.57; 95% CI: 0.44–0.73; *P* < 0.001), regardless of *ESR1*m status. At the prespecified interim overall survival (OS) analysis with a median follow up of 28.5 months, imlunestrant demonstrated a clinically significant longer median OS versus SOC ET (difference of 11.4 months; HR 0.60, 95% CI 0.43–0.86, *P* = 0.0043) in patients with *ESR1*m tumors, which did not cross the prespecified boundary for statistical significance. In all patients, median OS was not reached in the imlunestrant + abemaciclib arm and was 34.4 months in the imlunestrant arm^18^. Imlunestrant is now approved by the United States Food and Drug Administration for patients with ER+, HER2-, *ESR1*m ABC with disease progression following at least one line of ET^19^. While clinical efficacy is a key factor for both patients and clinicians, it is critical to characterize safety to inform treatment decisions, especially in the case of a novel therapeutic.

Here, we present a comprehensive safety analysis for the EMBER-3 study, characterizing the most common and clinically significant adverse events (AEs) and providing additional data for select subgroups. This detailed analysis is intended to help clinicians make more clinically informed decisions for their patients when administering imlunestrant with or without abemaciclib.

## Results

### Study population

The safety population comprised 859 patients who received at least one dose of study treatment (imlunestrant, *n* = 327; SOC ET, *n* = 324 [fulvestrant, *n* = 292; exemestane, *n* = 32]; imlunestrant + abemaciclib, *n* = 208). Baseline characteristics of the study population were previously reported and were generally balanced across treatment arms^17^. The data cutoff date of the final PFS analysis (June 24, 2024) was used for this analysis. Within the safety population, 65 (20%), 43 (13%), and 75 (36%) patients in the imlunestrant, SOC ET, and imlunestrant + abemaciclib arms, respectively, remained on study treatment.

### Imlunestrant vs SOC ET

In the imlunestrant arm, the median duration on therapy (Q1–Q3) was 170 (64–338) days compared to 143 (84–281) days for those receiving fulvestrant and 175 (74–309) days for those receiving exemestane.

**Fig. 1:**
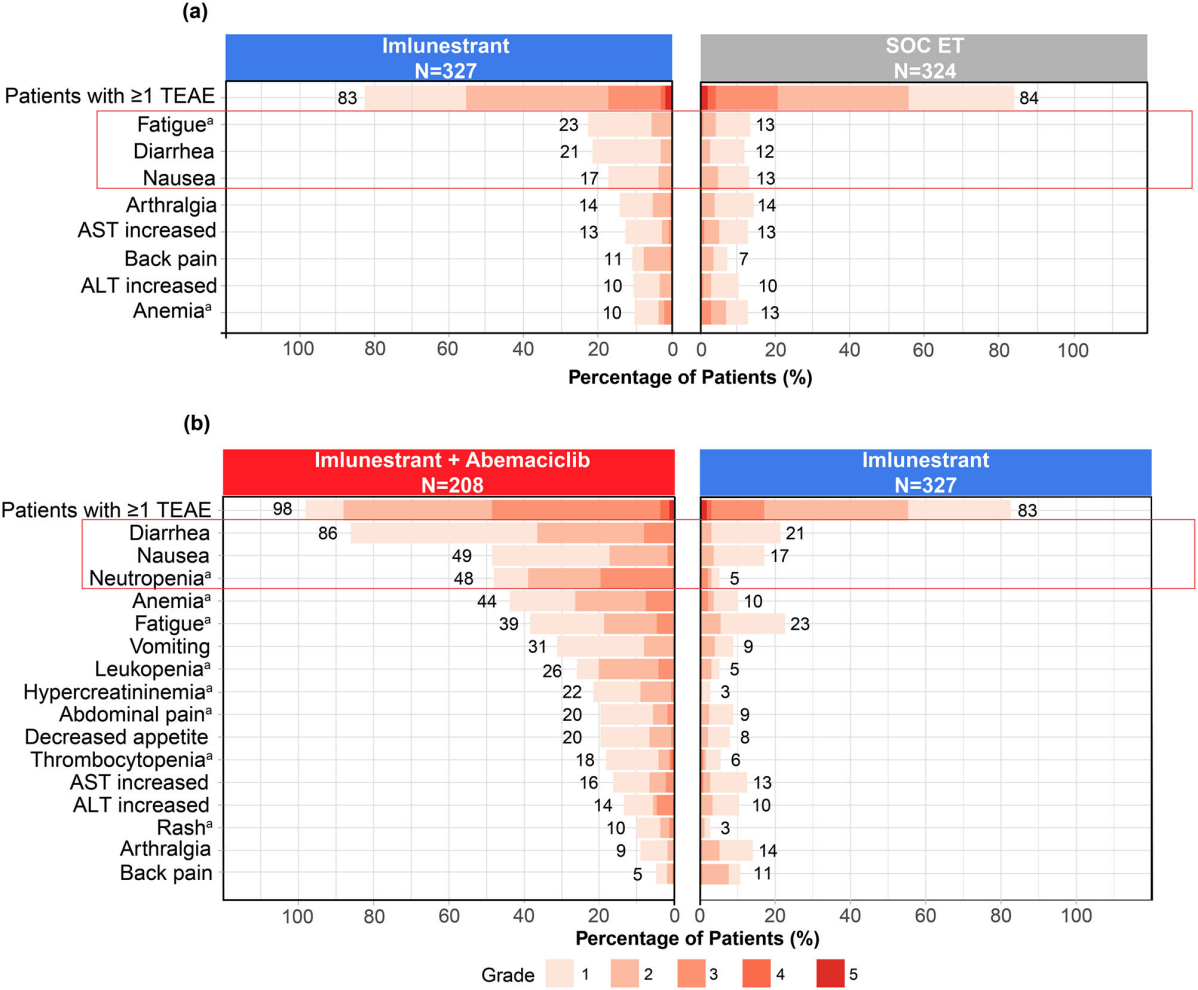
The most frequent TEAEs in ≥10% of patients in any treatment arm. **a** Imlunestrant monotherapy and SOC ET, and **b** imlunestrant + abemaciclib and imlunestrant monotherapy. ^a^Consolidated terms. ALT alanine aminotransferase, AST aspartate aminotransferase, N number of patients in the total safety population, SOC ET standard-of-care endocrine therapy, TEAE treatment-emergent adverse event.

The most common treatment-emergent AEs (TEAEs) with imlunestrant were fatigue, diarrhea, and nausea (Fig. 1A). Grade ≥3 TEAEs (imlunestrant, 17%; SOC ET, 21%) and treatment-emergent serious AEs (SAEs) (imlunestrant, 10%; SOC ET, 12%) were infrequent. Six patients (2%) in each arm died due to a TEAE during study treatment or within 30 days of treatment discontinuation (Supplementary Table 1).

### Characterization of the most frequent and clinically relevant TEAEs in the imlunestrant arm

Of the most common TEAEs reported, fatigue (23% vs 13%) and diarrhea (21% vs 12%) were more frequent with imlunestrant than with SOC ET, while the incidence of nausea was similar between the 2 arms (17% vs 13%). The majority of these TEAEs were grade 1 single episodes, occurred early, and rarely led to dose adjustments or discontinuation. The use of antidiarrheals (10% vs 7%) and antiemetics (10% vs 10%) was similar in both arms (Table 1).

**Table 1:** Characteristics of clinically relevant AEs for imlunestrant and SOC ET

TEAEs, n (%)	Imlunestrant (N=327)	SOC ET (N=324)
All events	270 (83)	273 (84)
Diarrhea	70 (21)	38 (12)
Grade 2 TEAE, n (%)	9 (3)	8 (3)
Grade ≥3 TEAE, n (%)	1 (0.3)	0
Patients with a single occurrence of TEAE, n (%)	54 (17)	34 (11)
Patients with >1 incidence of TEAE, n (%)	16 (5)	4 (1)
Median time to initial onset (Q1-Q3), days	30 (15-129)	52 (17-132)
Median duration of grade 2 TEAE (range), days	3 (1-28)	5 (1-55)
Dose interruptions/delays^a^, n (%)	2 (0.6)	0
Antidiarrheal medication in patients with diarrhea, n (%)	25 (36)	10 (26)
Antidiarrheal medication in the safety population, n (%)	33 (10)	23 (7)

Nausea	56 (17)	42 (13)
Grade 2 TEAE, n (%)	11 (3)	15 (5)
Grade ≥3 TEAE, n (%)	1 (0.3)	0
Patients with a single occurrence of TEAE, n (%)	48 (15)	38 (12)
Patients with >1 incidence of TEAE, n (%)	8 (2)	4 (1)
Median time to initial onset (Q1-Q3), days	20 (4-56)	57 (10-147)
Median duration of grade 2 TEAE (range), days	16 (4-89)	10 (1-90)
Dose reductions^b^, n (%)	1 (0.3)	0
Antiemetic medication in patients with nausea, n (%)	15 (27)	14 (33)
Antiemetic medication in the safety population, n (%)	32 (10)	33 (10)

Fatigue^c^	74 (23)	43 (13)
Grade 2 TEAE, n (%)	17 (5)	11 (3)
Grade ≥3 TEAE, n (%)	1 (0.3)	2 (0.6)
Patients with a single occurrence of TEAE, n (%)	66 (20)	42 (13)
Patients with >1 incidence of TEAE, n (%)	8 (2)	1 (0.3)
Median time to initial onset (Q1-Q3), days	42 (14-86)	29 (13-124)
Median duration of grade 2 TEAE (range), days	43 (7-529)	98 (9-232)
Dose interruptions/delays, n (%)	1 (0.3)	1 (0.3)
Dose reductions, n (%)	1 (0.3)	0
Dose discontinuations, n (%)	1 (0.3)	0

Elevated transaminases^d^	51 (16)	48 (15)
Grade 2 TEAE, n (%)	10 (3)	15 (5)
Grade ≥3 TEAE, n (%)	4 (1)	4 (1)
Patients with a single occurrence of TEAE, n (%)	24 (7)	19 (6)
Patients with >1 incidence of TEAE, n (%)	27 (8)	29 (9)
Median time to initial onset (Q1-Q3), days	58 (16-197)	43 (15-185)
Median duration of grade 2 TEAE (range), days	27 (11-97)	29 (5-86)
Dose interruptions/delays, n (%)	5 (2)	2 (0.7)
Dose reductions, n (%)	2 (0.6)	0
Dose discontinuations, n (%)	4 (1)	0

Dose adjustments of exemestane were not allowed. Administration of fulvestrant injections later than required per protocol was counted as a dose delay rather than dose interruption.

AE, adverse event; ALT, alanine aminotransferase; AST, aspartate aminotransferase; N, number of patients in the total safety population; n, number of patients in the specified category; Q, quartile; SOC ET, standard-of-care endocrine therapy; TEAE, treatment-emergent adverse event.

^a^There were no dose reductions or treatment discontinuations due to diarrhea in either arm

^b^There were no dose interruptions or treatment discontinuations due to nausea in either arm

^c^Fatigue includes fatigue and asthenia.

^d^Elevated transaminases includes increased ALT, increased AST, drug-induced liver injury, increased hepatic enzymes, hepatotoxicity, hypertransaminasemia, and increased transaminases.

Transaminase elevations were infrequent, primarily grade 1, led to few dose modifications, and were comparable between arms (16% with imlunestrant vs 15% with SOC ET). Furthermore, grade ≥3 events were rare, short in duration (median [range], 5 [4–27] days with imlunestrant), and mostly single occurrences (Table 1). Concurrent increases in transaminases and total bilirubin were infrequent, and only 3 patients receiving imlunestrant had postbaseline alanine aminotransferase/aspartate aminotransferase increases >3× upper limit of normal (ULN) and total bilirubin increases >2× ULN; all 3 patients had alkaline phosphatase >3× ULN and liver metastases. No cases fulfilled Hy’s law criteria for liver injury.

### Dose adjustments and discontinuations in the imlunestrant arm

Dose interruptions and reductions due to TEAEs were low in frequency (10% and 2%, respectively) and occurred throughout the treatment period. The most common reasons for dose interruptions included vomiting and transaminase elevations (2% each), with transaminase elevations (1%) being the primary reason for dose reductions (Supplementary Table 2).

Discontinuations due to TEAEs were infrequent (4.6% in the imlunestrant arm, 2.8% due to nonfatal AEs, and 1.8% due to fatal AEs vs 1.2% in the SOC ET arm, all of which were due to fatal AEs) (Supplementary Table 1). Although low in frequency, transaminase elevations were the only TEAE resulting in treatment discontinuation in more than 1 patient (Supplementary Table 2).

### Imlunestrant + abemaciclib

In the imlunestrant + abemaciclib arm, the median (Q1–Q3) duration on treatment was 233 (92–393) days on abemaciclib and 235 (105–394) days on imlunestrant.

The most frequently reported TEAEs were diarrhea, nausea, and neutropenia, which were mostly grades 1 or 2 (Fig. 1B). There were 48.6% of patients who experienced grade 3 TEAEs, and the most common grade 3 event was neutropenia. The incidence of treatment-emergent SAEs was low (17%).

Three patients (1%) died due to a TEAE during treatment or within 30 days of discontinuation of imlunestrant + abemaciclib (Supplementary Table 1).

### Characterization of the most frequent and clinically relevant TEAEs in the imlunestrant + abemaciclib arm

#### Diarrhea

**Fig. 2:**
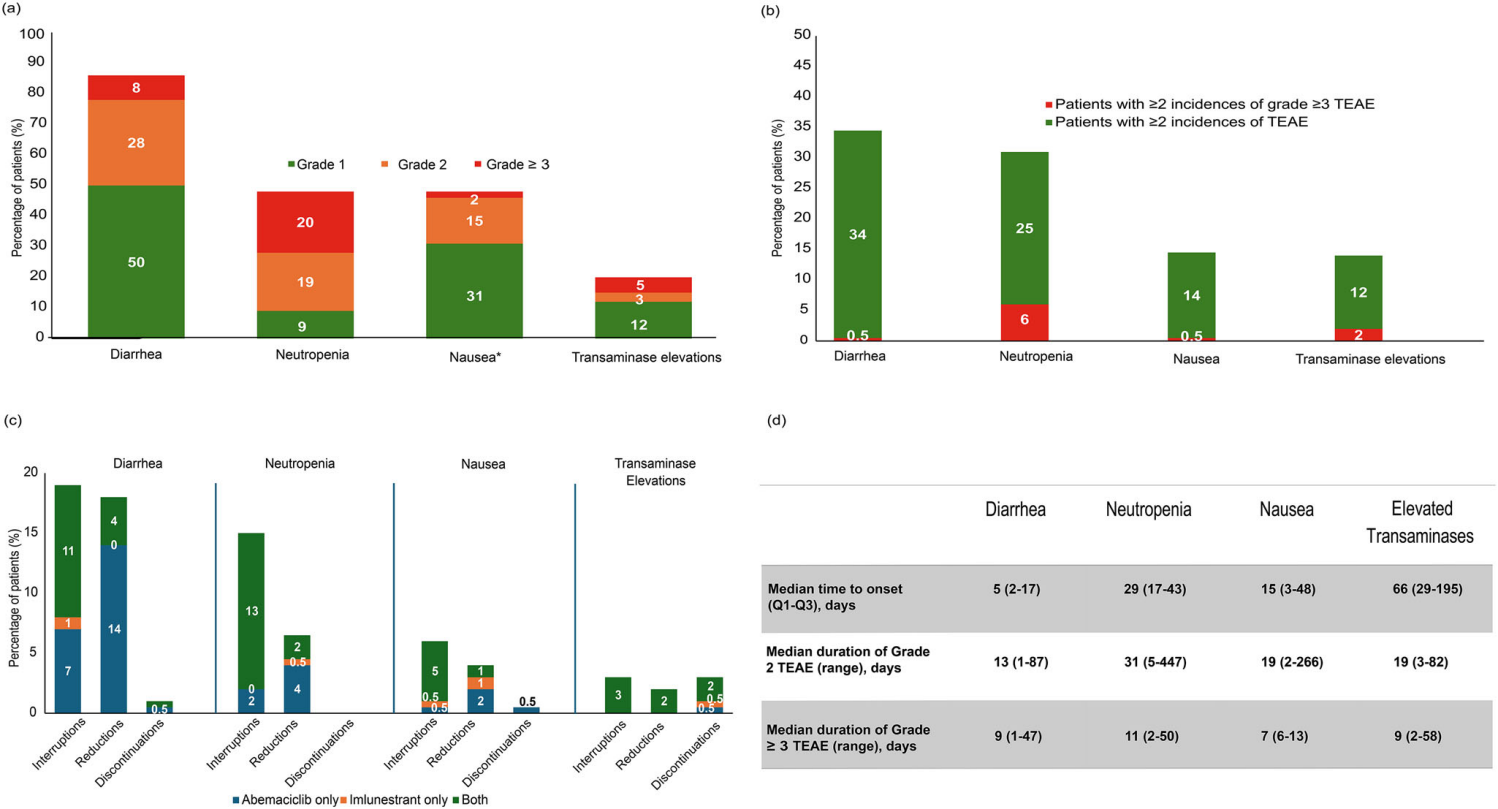
Characteristics of the clinically relevant TEAEs for imlunestrant + abemaciclib (*n* = 208). **a** Grades of clinically relevant TEAEs; **b** Percentages of patients with ≥2 incidences of clinically relevant TEAEs; **c** Dose modifications and discontinuations by drug due to clinically relevant TEAEs; **d** Median time to onset and duration of clinically relevant AEs. *The total is 49% rather than 48% due to rounding. Neutropenia includes neutropenia and neutrophil count decreased. AE adverse event, N number of patients in the total safety population, n number of patients in the specified category, Q quartile, TEAE treatment-emergent adverse event.

**Fig. 3:**
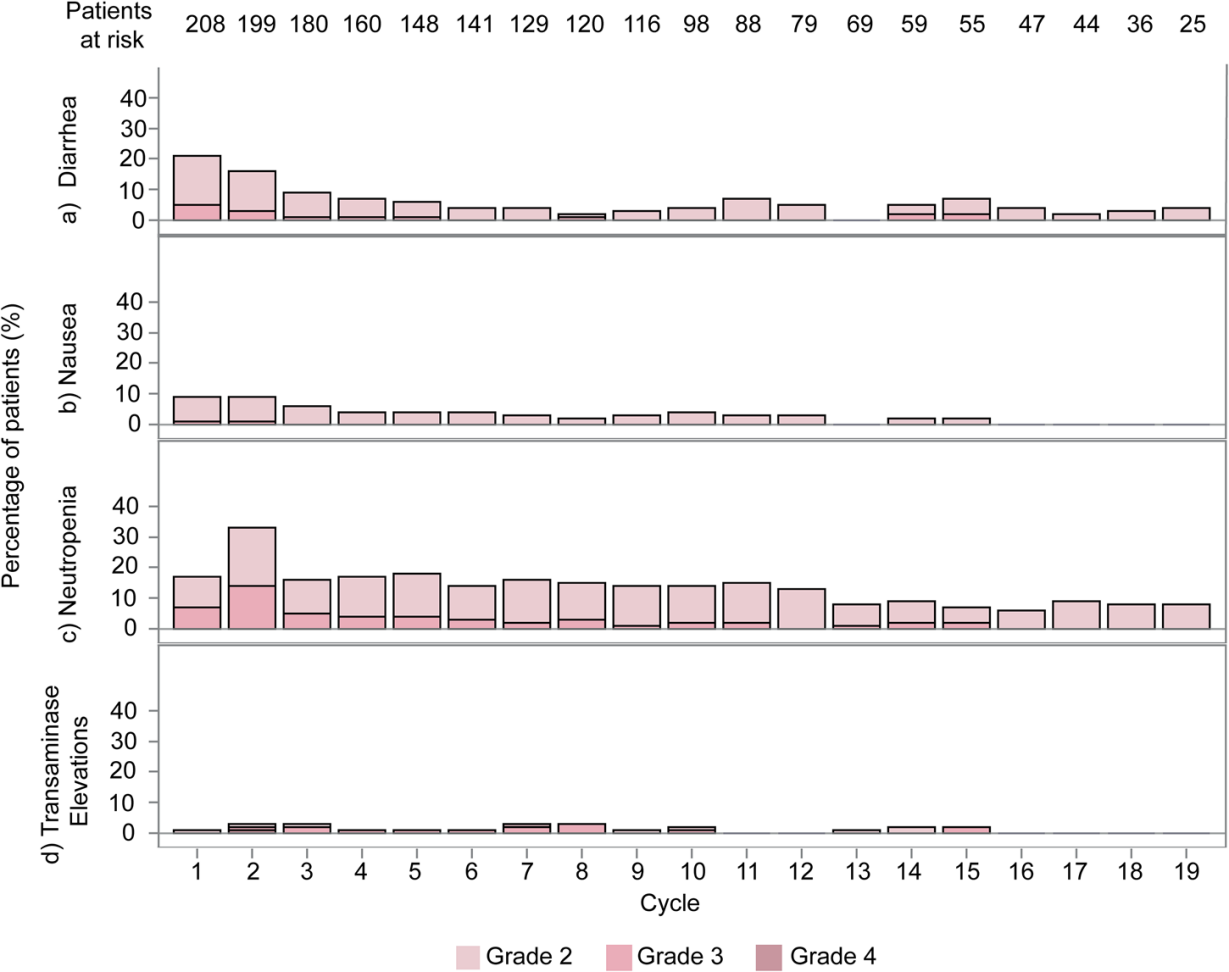
Percentage of clinically relevant TEAEs over time in the imlunestrant + abemaciclib arm. **a** Diarrhea; **b** nausea; **c** neutropenia; **d** transaminase elevations. TEAE treatment-emergent adverse event.

Diarrhea was common in the imlunestrant + abemaciclib arm (86%) but was predominantly low grade and generally occurred early in treatment (median time to onset, 5 days) (Fig. 2). Grade ≥3 events occurred in 8% of patients, primarily in the first 3 months, and were of short duration (median, 9 days) (Figs. 2 and 3). SAEs of diarrhea were reported in 2 (1%) patients and required hospitalizations. Diarrhea was effectively managed with antidiarrheal medication (68%) and dose modifications (dose reductions, 18%; interruptions, 19%); discontinuations were rare, occurring in only 2 patients—one discontinued both drugs and one discontinued abemaciclib while continuing imlunestrant (Fig. 2).

#### Neutropenia

Neutropenia was the most frequently reported grade ≥3 AE (20%) in the imlunestrant + abemaciclib arm (Fig. 2). Grade ≥3 events were mostly single occurrences, and median duration was 11 days. There were no SAEs of neutropenia reported. Neutropenia was generally managed with dose adjustments (dose interruptions, 15%; dose reductions, 7%), and no patient discontinued treatment due to neutropenia. One patient experienced febrile neutropenia.

#### Nausea

Nausea was reported in 49% of the patients in the imlunestrant + abemaciclib arm, was predominantly grade 1, and generally occurred early in treatment (median time to onset, 15 days) (Fig. 2). Grade ≥3 events were infrequent (2%) and typically occurred within the first 3 months (Fig. 3). These events were short-lived, with a median duration of 7 days, and were effectively managed with supportive care (antiemetics were administered to 21% of patients) (Fig. 2).

#### Elevated transaminases

Transaminase elevations were reported in 20% of patients in the imlunestrant + abemaciclib arm, with grade ≥3 events reported in 5% of patients (Fig. 2). Grade ≥3 events were of short duration (median, 9 days), with 2% of patients experiencing ≥2 occurrences, and events were managed with dose adjustments. Though transaminase elevations were the most common TEAE leading to study treatment discontinuation, the discontinuation rate was low (2% for both drugs; 0.5% for imlunestrant only; 0.5% for abemaciclib only). Three patients had postbaseline alanine aminotransferase/aspartate aminotransferase increases >3× ULN and total bilirubin increases >2× ULN. All 3 patients had alkaline phosphatase >2× ULN and confounding factors. One patient had liver metastasis, one had a bile duct stone, and the remaining patient had hepatic steatosis. No cases fulfilling Hy’s law criteria for liver injury were identified.

#### Dose adjustments and discontinuations in the imlunestrant + abemaciclib arm

**Fig. 4:**
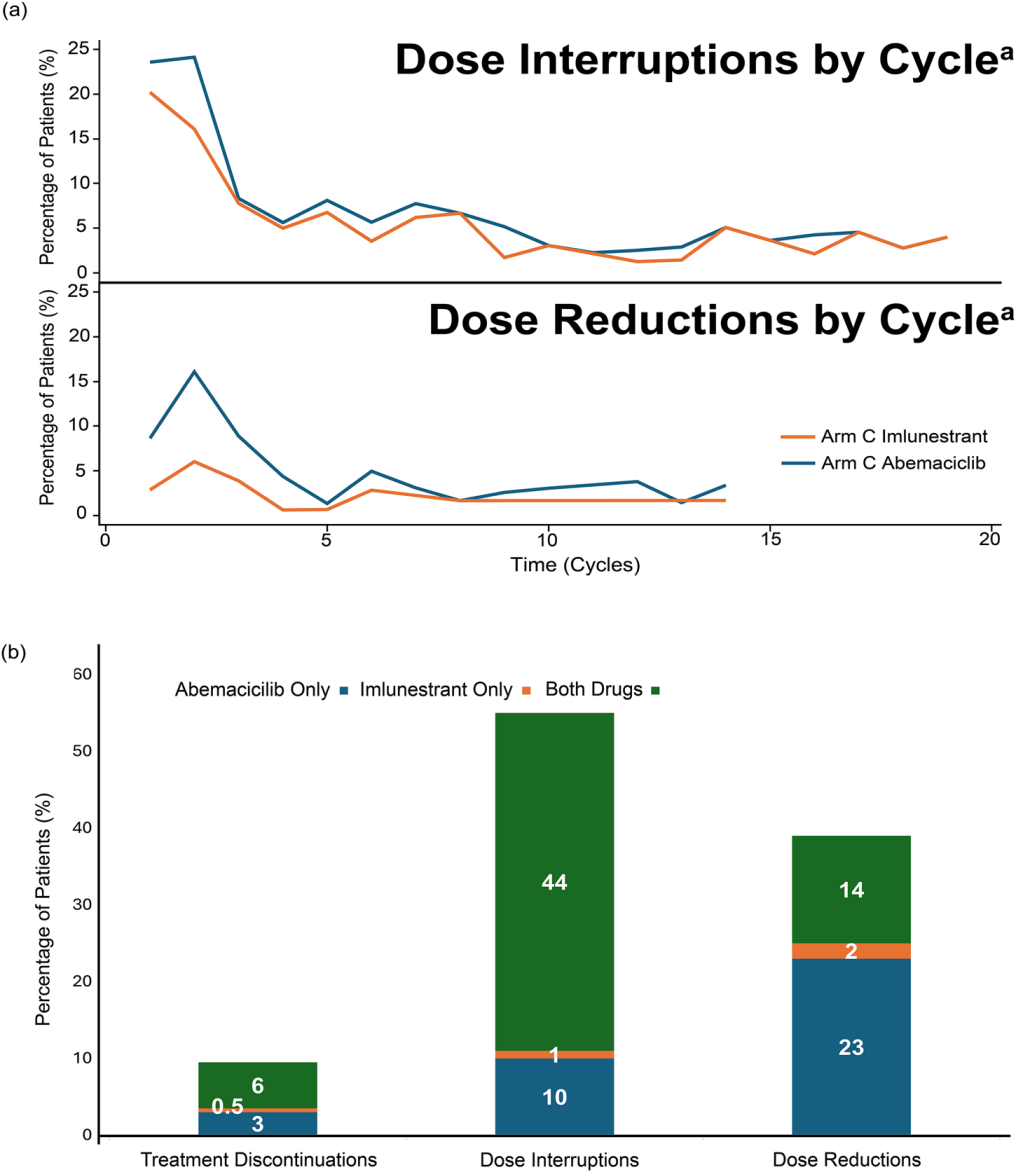
Treatment modifications and discontinuations due to TEAEs in the imlunestrant + abemaciclib arm. **a** Dose adjustments over time due to TEAEs; **b** Treatment discontinuations and adjustments by drug due to TEAEs. ^a^The standard-of-care endocrine therapy and imlunestrant monotherapy arms are not shown. No dose adjustment was allowed for exemestane per protocol. Dose delays for fulvestrant occurred in 7% of patients. TEAE treatment-emergent adverse event.

Dose interruptions and reductions due to TEAEs occurred in 55% and 39% of patients, respectively, and were most commonly due to diarrhea (19% and 18%, respectively). Overall, 61% of patients experienced any dose adjustments (Supplementary Table 2). Most dose reductions involved only abemaciclib, while dose interruptions typically involved both study drugs, with most adjustments occurring within the first 4 months of therapy (Fig. 4).

In the imlunestrant + abemaciclib arm, 6.3% of patients discontinued both drugs due to TEAEs (5.3% due to nonfatal and 1.0% due to fatal AEs); additionally, 3% of patients discontinued only abemaciclib and 0.5% discontinued only imlunestrant (Fig. 4).

#### Other TEAEs of interest for abemaciclib

Infections, venous thromboembolism (VTE), and interstitial lung disease (ILD) are known AEs of special interest for abemaciclib^20^. Infections were reported in 31% of patients in the imlunestrant + abemaciclib arms and were mostly low grade (grade ≥3 events, 4%, including one grade 5 event). Dose interruptions and reductions due to infection were required in 6% and 1% of patients, respectively. One patient discontinued study treatment due to a fatal infection event.

The incidences of VTE and ILD were low (3% and 2%, respectively), with a single grade 3 VTE (0.5%) and no grade 3 ILD events. No VTEs or ILDs resulted in treatment discontinuations.

#### Other TEAEs of interest in the SERD class

Photopsia, observed with some oral SERDs, was not reported in either of the imlunestrant-containing arms. Prolonged QTc was observed in 1%, 0%, and 2% of patients in the imlunestrant, SOC ET, and imlunestrant + abemaciclib arms, respectively. Bradycardia and dyslipidemia were infrequent and predominately low grade (bradycardia: 2%, 0%, and 1%; dyslipidemia: 7%, 9%, and 8% in the imlunestrant, SOC ET, and imlunestrant + abemaciclib arms, respectively), with only 1 case of grade 3 hypertriglyceridemia reported in the imlunestrant arm.

Among patients without prior lipid-modifying therapy, few initiated treatment during the study (imlunestrant, 6%; SOC ET, 4%; imlunestrant + abemaciclib, 2%).

In patients with available postbaseline cholesterol measurements, increases from low/normal to high were more frequent for high-density lipoprotein than low-density lipoprotein levels (imlunestrant, 16% vs 1%; SOC ET, 11% vs 2%; imlunestrant + abemaciclib, 38% vs 1%).

#### Safety by age subgroup

In EMBER-3, the median age was 61 years (range, 27-89). In the analyses of patients under and over 65 years of age, the incidence of any-grade TEAEs and SAEs was similar between age groups, although some differences were observed in some individual TEAEs in the imlunestrant + abemaciclib arm (Table 2). Patients >75 years of age comprised 11%, 15%, and 13% of the patients in the imlunestrant, SOC ET, and imlunestrant + abemaciclib arms, respectively. In the imlunestrant + abemaciclib arm, patients ≥75 years of age had more dose reductions and discontinuations due to AEs than patients 65–74 years of age (Supplementary Table 3).

**Table 2:** Safety by age

Relevant TEAEs, *n* (%)		Imlunestrant (*N* = 327)		Imlunestrant + abemaciclib (*N* = 208)	
		<65 years (*n* = 209)	≥65 years (*n* = 118)	<65 years (*n* = 118)	≥65 years (*n* = 90)
**Patients with** ≥ **1 TEAE**		**172 (82)**	**98 (83)**	**115 (98)**	**89 (99)**
**Patients with** ≥ **1 grade** ≥ **3 TEAE**		**40 (19)**	**16 (14)**	**54 (46)**	**47 (52)**
Diarrhea	Any grade	45 (22)	25 (21)	102 (86)	77 (86)
	Grade ≥3	0	1 (0.8)	7 (6)	10 (11)
Fatigue^a^	Any grade	44 (21)	30 (25)	43 (36)	37 (41)
	Grade ≥3	1 (0.5)	0	2 (2)	8 (9)
Nausea	Any grade	34 (16)	22 (19)	50 (42)	51 (57)
	Grade ≥3	1 (0.5)	0	3 (3)	1 (1)
AST increased	Any grade	30 (14)	11 (9)	20 (17)	14 (16)
	Grade ≥3	2 (1.0)	1 (0.8)	3 (3)	2 (2)
ALT increased	Any grade	25 (12)	9 (8)	14 (12)	14 (16)
	Grade ≥3	1 (0.5)	0	4 (3)	6 (7)
Anemia^a^	Any grade	20 (10)	13 (11)	49 (42)	42 (47)
	Grade ≥3	7 (3)	0	9 (8)	7 (8)
Vomiting	Any grade	18 (9)	11 (9)	33 (28)	32 (36)
	Grade ≥3	1 (0.5)	1 (0.8)	0	1 (1)
Abdominal pain^a^	Any grade	18 (9)	11 (9)	27 (23)	14 (16)
	Grade ≥3	1 (0.5)	0	4 (3)	0
Neutropenia^a^	Any grade	12 (6)	5 (4)	61 (52)	39 (43)
	Grade ≥3	6 (3)	1 (0.8)	24 (20)	17 (19)
Leukopenia^a^	Any grade	13 (6)	4 (3)	29 (25)	25 (28)
	Grade ≥3	2 (1)	0	6 (5)	3 (3)
Hypercreatininemia^a^	Any grade	2 (1)	7 (6)	24 (20)	21 (23)
	Grade ≥3	0	1 (0.8)	0	2 (2)
Patients with ≥1 treatment-emergent SAE^b^		24 (12)	10 (9)	19 (16)	16 (18)
Discontinuations due to AE^b^		9 (4)	5 (4)	5 (4)	8 (9)
Dose reductions due to AE		5 (2)	3 (3)	38 (32)	44 (49)
Dose withheld/delayed due to AE		23 (11)	11 (9)	58 (49)	57 (63)

The most frequent TEAEs in ≥15% of patients in the imlunestrant arms among patients <65 and ≥65 years of age are shown.

*AE* adverse event, *ALT* alanine aminotransferase, *AST* aspartate aminotransferase, *N* number of patients in the total safety population, *n* number of patients in the specified category, *Q* quartile, *SOC ET* standard-of-care endocrine therapy, *TEAE* treatment-emergent adverse event.

^a^Consolidated terms.

^b^Deaths were also included as SAEs and discontinuations due to AEs.

#### Safety in the *ESR1*m subgroup

In EMBER-3, 42%, 36%, and 32% of patients had *ESR1*m ABC in the imlunestrant, SOC ET, and imlunestrant + abemaciclib arms, respectively. The median duration of treatment was similar to that in the overall safety population for each arm (Supplementary Table 4).

Although a higher incidence of TEAEs was noted in patients with *ESR1*m receiving imlunestrant or imlunestrant + abemaciclib than in the respective overall safety populations (imlunestrant, 90% vs 83%; SOC ET, 80% vs 84%; imlunestrant + abemaciclib, 99% vs 98%), this difference was not clinically relevant, as demonstrated by the similar incidences of grade ≥3 AEs, SAEs, deaths due to AEs, and discontinuations due to AEs between patients with *ESR1*m and the overall safety populations for these treatment arms (Supplementary Tables 1, 2, and 4; Fig. 1).

## Discussion

This large phase III trial of 874 patients allowed for robust characterization of the safety profile of imlunestrant, a novel oral SERD, as monotherapy and in combination with abemaciclib in patients with ER + , HER2- ABC. While the overall safety has been previously described^17^, the present analysis offers a more detailed characterization.

Among patients receiving imlunestrant monotherapy, the incidences of most TEAEs were similar to those in patients receiving SOC ET, with a slightly lower overall incidence of grade ≥3 TEAEs. Although fatigue and diarrhea were more frequent with imlunestrant than with SOC ET, they were predominantly grade 1, single events requiring few dose adjustments and little to no treatment discontinuations. Use of supportive medication (antiemetics and antidiarrheals) was comparable between imlunestrant and SOC ET arms, suggesting minimal additional treatment burden for the incorporation of imlunestrant monotherapy into current clinical practice. In addition, its oral administration provided a practical advantage over the monthly intramuscular injections required with fulvestrant.

Although cross-trial comparisons should be interpreted with caution, imlunestrant use was associated with low or no incidence of clinically relevant toxicities that are observed with other members of the SERD class, including bradycardia, photopsia, prolonged QTc, and dyslipidemia^12,14,21–23^. These findings further support a lower treatment burden for patients and healthcare professionals with respect to screening and monitoring requirements when using imlunestrant.

The safety profile for the imlunestrant + abemaciclib arm was consistent with the established abemaciclib safety profile from prior studies evaluating the abemaciclib + fulvestrant combination (Supplementary Table 5). No new toxicities were observed^24–27^. The most common TEAEs, including diarrhea, nausea, and neutropenia, were generally low grade, occurred early, and were effectively managed with supportive care and dose adjustments. Discontinuation rates were consistent with findings from the postMONARCH trial and numerically lower than those from the MONARCH 2 trial, which may reflect improved clinical management of abemaciclib over time^6,25^.

In the subgroup analysis by age, low rates of discontinuations and adjustments due to AEs indicated that imlunestrant monotherapy was generally well tolerated, with similar incidences of TEAEs in both younger and older patients (<65 and ≥65 years of age). Among patients 65 years of age and older treated with imlunestrant + abemaciclib, numerically higher incidences of some low-grade TEAEs (eg, fatigue and nausea) were observed, likely reflecting the increased susceptibility of this population. Similar differences between age groups have been previously reported for abemaciclib^28^. Among patients over 75 years of age treated with imlunestrant monotherapy, the low rates of discontinuations and adjustments due to AEs indicated that imlunestrant monotherapy was a tolerable treatment option.

In the subgroup of patients with *ESR1*m ABC, TEAEs were generally consistent with those in the overall safety population (especially grade ≥3 TEAEs, SAEs, and deaths due to AEs), with some differences in the incidences of individual TEAEs.

Limitations of this study include the open-label nature of the study, which introduced some potential for bias in AE reporting; however, the sizeable patient population and methods of collecting and classifying TEAEs were designed to minimize this bias. The protocol recommended open-ended, non-leading verbal questioning of the participant to inquire about AE occurrences, which minimized reporting bias by avoiding symptom prompting and allowing unsolicited AE reporting. Standardized collection procedures, objective coding criteria, staff training, and independent review of AE classification were also essential to ensure consistency and reliability of safety data. Additionally, access to study data was strictly restricted prior to the final analyses, and the sponsor and investigative sites remained blinded to aggregate results. Scrambled treatment assignments were used for independent Data Monitoring Committee oversight of interim safety and efficacy analyses. Additionally, subgroup analyses were limited by smaller patient numbers. This analysis primarily describes short-term safety. In a later pre-specified EMBER-3 OS analysis, with more than a year of additional follow-up, no new safety signals were observed, and the safety profile of both monotherapy and the combination remained consistent with the primary datacut^18^. Safety monitoring continues in EMBER-3. Additionally, the long-term safety and efficacy of imlunestrant are being evaluated in ongoing studies, including in trials in early breast cancer (NCT05514054 and NCT07287098)^29,30^.

In conclusion, imlunestrant monotherapy demonstrated a favorable safety profile, with the most common TEAEs being grade 1 and requiring few dose adjustments or discontinuations. While acknowledging the limitations of cross-trial comparisons, the safety profile of imlunestrant appears favorable, and its use was not associated with toxicities observed with other SERDs in development, such as photopsia, bradycardia, and QTc prolongation^12,14^. The combination of imlunestrant and abemaciclib demonstrated a safety profile consistent with the known toxicities associated with abemaciclib use, without any new safety findings. The most common AEs were generally manageable with supportive care and standard dose modifications. The superior efficacy of imlunestrant monotherapy in patients with *ESR1*m tumors and the enhanced efficacy of the imlunestrant + abemaciclib combination in patients regardless of *ESR1*m status, combined with a tolerable safety profile, support the use of imlunestrant both as monotherapy and in combination in this clinical setting.

## Methods

### Study design and patient population

The EMBER-3 trial (NCT04975308) is an open-label, multicenter, randomized, phase III trial that analyzed the efficacy and safety of imlunestrant monotherapy, SOC ET, or imlunestrant + abemaciclib in patients with ER+, HER2- ABC with disease recurrence or progression during or after either (neo)adjuvant treatment or first-line treatment for ABC with an AI with or without a CDK4/6i. No other prior therapy was permitted in the advanced setting^17^.

The protocol was approved by the ethical review board at each site (Supplementary Table 6). The trial was conducted in accordance with the principles of the Declaration of Helsinki. All participants provided written informed consent.

### Treatment

The detailed study design has been previously published^17^, and patients were randomly assigned 1:1:1 to receive imlunestrant (400 mg once daily [QD]) or investigator’s choice of SOC ET (oral exemestane [25 mg QD] or fulvestrant [500 mg intramuscular injection on days 1 and 15 of cycle 1 and on day 1 of subsequent 28-day cycles]) or imlunestrant (400 mg QD) + abemaciclib (150 mg twice daily [BID]). Treatment continued until disease progression or unacceptable toxicity. Dose adjustments (interruptions and reductions) for imlunestrant and abemaciclib were required per protocol to manage TEAEs of specified severity (Supplementary Fig. 1). A maximum of 1 dose reduction for imlunestrant (200 mg QD) and 2 dose reductions (100 mg and 50 mg BID) for abemaciclib were allowed. Additional dose reductions required discontinuation of the respective study drug. In the imlunestrant + abemaciclib arm, if either study drug was discontinued, the other could be continued. Dose adjustments of exemestane were not allowed. Fulvestrant dose adjustments were determined by the investigator. Supportive care was per investigator discretion. Patients in all arms were instructed to initiate antidiarrheal therapy at the onset of loose stools.

### Safety assessment

The type, severity, seriousness, duration, and relatedness of TEAEs to study treatment were collected throughout the study. Severity was assessed according to the National Cancer Institute Common Terminology Criteria for Adverse Events, version 5.0. Local and central laboratory assessments—including hematology and clinical chemistry—were performed at baseline, on day 1, every 2 weeks during the first 2 months (for imlunestrant arms), monthly thereafter during treatment, and approximately 30 days after treatment discontinuation. Other assessments (eg, hormone levels, lipid panels, coagulation) were conducted at predefined or clinically indicated intervals per protocol.

### Statistical analysis

The safety population included patients who received at least one dose of any study treatment. Each patient was analyzed according to the treatment received.

TEAEs were summarized by maximum toxicity regardless of causality. Clinically synonymous terms were grouped together under a consolidated term^17^. Descriptive analyses characterized the most common and clinically relevant TEAEs in terms of severity, timing, duration, and their management. Treatment-emergent SAEs and deaths were summarized. A detailed analysis of discontinuations and dose adjustments due to TEAEs was conducted, including main reasons, timing, and analysis by drug in the combination arm. The differences in safety profile in age subgroups (<65 and ≥65 years; and <65, 65-74, and ≥75 years) and in the *ESR1*m subgroup were explored. All statistical analyses were performed using SAS (version 9.4 or higher) or R (version 4.4.2 or higher) software.

## Supplementary information

EMBER-3Safety_Supplement

## Data Availability

Lilly provides access to all individual participant data collected during the trial, after anonymization, with the exception of pharmacokinetic or genetic data. Data are available to request 6 months after the indication studied has been approved in the US and EU and after primary publication acceptance, whichever is later. No expiration date of data requests is currently set once data are made available. Access is provided after a proposal has been approved by an independent review committee identified for this purpose and after receipt of a signed data sharing agreement. Data and documents, including the study protocol, statistical analysis plan, clinical study report, and blank or annotated case report forms, will be provided in a secure data sharing environment. For details on submitting a request, see the instructions provided at www.vivli.org.
