# Supplementary material for: Imlunestrant with or without abemaciclib in advanced breast cancer: safety analyses from the EMBER-3 trial
Source: NPJ Breast Cancer. 2026 Apr 27;12:88. doi: 10.1038/s41523-026-00950-z (PMC13324159; doi:10.1038/s41523-026-00950-z)
Supplement: Supplementary file 1 — EMBER-3Safety_Supplement [file 41523_2026_950_MOESM1_ESM.docx]

**SUPPLEMENTARY MATERIAL**

**Imlunestrant with or without abemaciclib in advanced breast cancer: safety analyses from the EMBER-3 trial**

Joyce O’Shaughnessy, M.D.^1^, Francois-Clement Bidard, M.D.^2^, Patrick Neven, M.D.^3^, Monica Lis Casalnuovo, M.D.^4^, Philippe Aftimos, M.D.^5^, Cristina Saura, M.D.^6^, Nadia Harbeck, M.D.^7^, Lisa A. Carey, M.D.^8^, Giuseppe Curigliano, M.D.^9,10^, Jose A Garcia-Saenz, M.D.^11^, Maria Fernandez Abad, M.D.^12^, Larissa de Paula, M.D.^13^, Yeon Hee Park, M.D.^14^, Ozgur Ozyilkan, M.D.^15^, Maria Munoz, Ph.D^16^, Emily Barrett, M.Sc^16^, Shanshan Cao, Ph.D^16^, Aarti Chawla, Ph.D^16^, Komal L. Jhaveri, M.D.^17,18^

^1^Baylor University Medical Center, Texas Oncology, Sarah Cannon Research Institute, Dallas, TX, USA

^2^Institut Curie, Paris and Saint Cloud, France

^3^Department of Oncology, Universitaire Ziekenhuizen Leuven, Leuven, Belgium

^4^Hospital María Curie, Buenos Aires, Argentina

^5^Institut Jules Bordet, Hôpital Universitaire de Bruxelles (HUB), Brussels, Belgium

^6^Vall d’Hebron University Hospital, Vall d’Hebron Institute of Oncology (VHIO), Barcelona, Spain

^7^Breast Center, Department of Obstetrics and Gynecology and CCC Munich, LMU University Hospital, Munich, Germany

^8^University of North Carolina at Chapel Hill, Chapel Hill, NC, USA

^9^Department of Oncology and Hemato-Oncology, University of Milano, Milano, Italy

^10^European Institute of Oncology, IRCCS, Milano, Italy

^11^Hospital Clinico Universitario San Carlos, Madrid, Spain

^12^Hospital Universitario Ramón y Cajal, Madrid, Spain

^13^Núcleo de Pesquisa do Instituto Brasileiro de Controle do Câncer (IBCC Oncologia), São Paulo, Brazil

^14^Samsung Medical Center, Sungkyunkwan University School of Medicine, Seoul, Republic of Korea

^15^Baskent University, Adana, Turkey

^16^Eli Lilly and Company, Indianapolis, IN, USA

^17^Memorial Sloan Kettering Cancer Center, NY, USA

^18^Weill Cornell Medical College, NY, USA

**
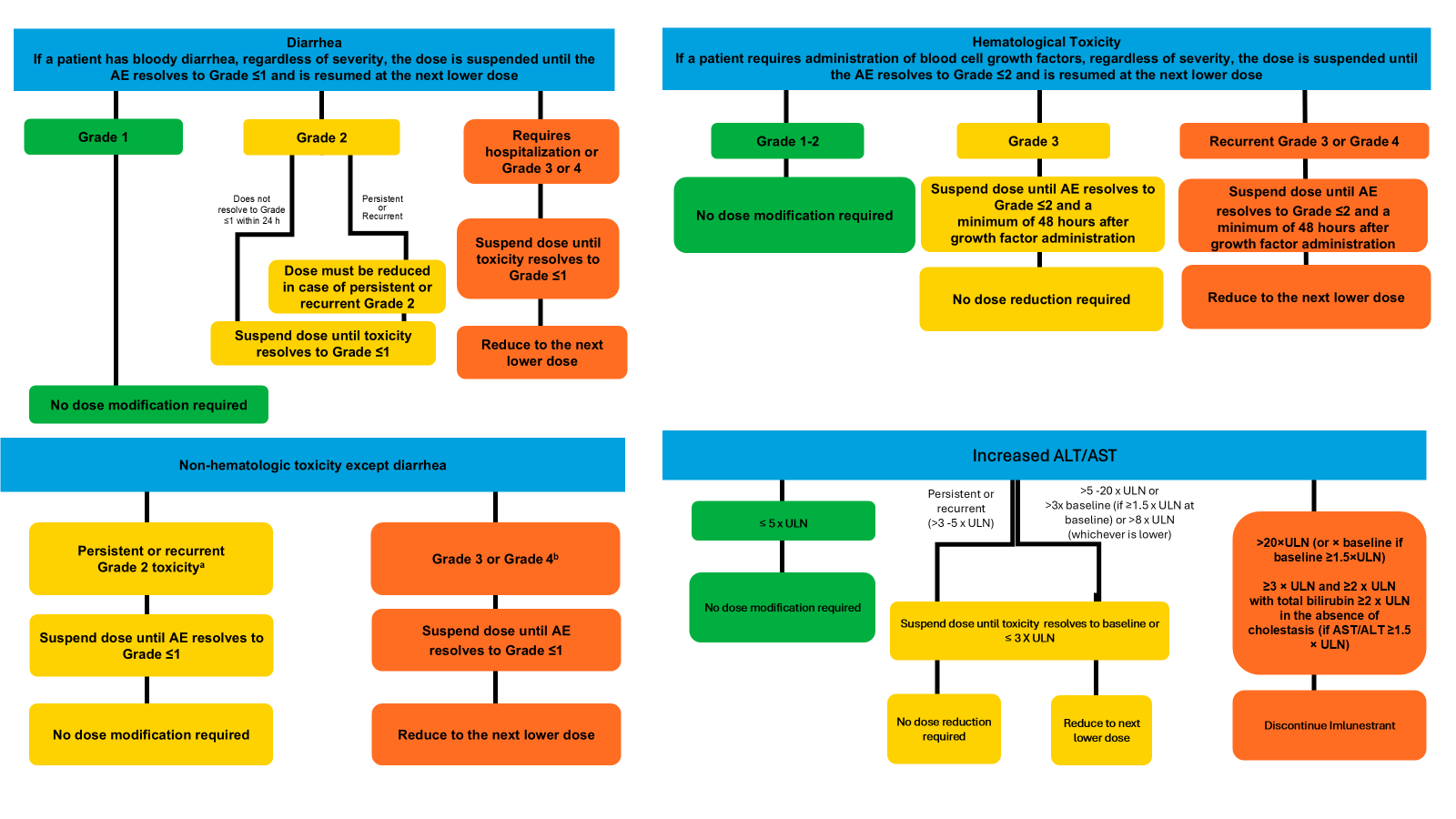
**

**Supplementary Fig. 1.** **Protocol requirements for dose adjustments of imlunestrant.** ^a^That does not resolve with maximal supportive measurement within 7 days to baseline or grade 1; ^b^Except for nonhepatic asymptomatic laboratory changes. AE, adverse event; ALT, alanine aminotransferase; AST, aspartate aminotransferase; h, hours; TBL, total bilirubin; ULN, upper limit of normal.

**Supplementary Table 1 |** **Overview of TEAEs leading to death**

| **Relevant TEAEs, n (%)** | **Imlunestrant**  **(N=327)** | **SOC ET**  **(N=324)** | **Imlunestrant + abemaciclib**  **(N=208)** |
| --- | --- | --- | --- |
| **Patients who died due to TEAEs during or within 30 days of treatment discontinuation** | **6 (1.8)^a^** | **6 (1.9)** | **3 (1.4)** |
| Cardiac arrest | 1 (0.3) | 2 (0.6) | 0 |
| Acute myocardial infarction | 1 (0.3) | 0 | 0 |
| Hypovolaemic shock | 1 (0.3) | 0 | 0 |
| Right ventricular failure | 1 (0.3)^b^ | 0 | 0 |
| Upper gastrointestinal hemorrhage | 1 (0.3) | 0 | 0 |
| Death (unknown cause) | 0 | 1 (0.3) | 1 (0.5)^b^ |
| Myocardial infarction | 0 | 0 | 1 (0.5) |
| Pneumonia | 0 | 0 | 1 (0.5) |
| Abdominal infection | 0 | 1 (0.3) | 0 |
| Blood calcium increased | 0 | 1 (0.3) | 0 |
| Chronic kidney disease | 0 | 1 (0.3) | 0 |

N, number of patients in the total safety population; n, number of patients in the specified category; SOC ET, standard-of-care endocrine therapy; TEAE, treatment-emergent adverse event.

^a^One patient in the imlunestrant arm died due to study disease but also had a TEAE of septic shock due to dengue infection that was considered fatal by the investigator.

^b^Considered by the investigator to be related to the study drug, but there was insufficient evidence of a causal association due to significant medical history and other confounding factors.

**Supplementary Table 2 | Dose discontinuations and adjustments due to TEAEs**

|  | Imlunestrant  (N=327) | SOC ET  (N=324) | Imlunestrant + abemaciclib  (N=208) |
| --- | --- | --- | --- |
| **Treatment discontinuations due to TEAEs, n (%)^a^** | **15 (5)**^b^ | **4 (1)** | **13 (6)** |
| Transaminase elevations^c^ | 4 (1) | 0 | 4 (2) |
| Cardiac arrest | 1 (0.3) | 2 (0.6) | 0 |
| **Dose adjustments due to TEAEs, n (%)** | **34 (10)** | **23 (7)** | **126 (61)** |
| **Dose interruptions/delays due to TEAEs, n (%)** | **34 (10)** | **21 (7)** | **115 (55)** |
| Transaminase elevations^c^ | 5 (2) | 2 (0.7) | 6 (3) |
| Vomiting | 5 (2) | 0 | 5 (2) |
| Neutropenia^c^ | 3 (0.9) | 0 | 31 (15) |
| COVID-19^c^ | 3 (0.9) | 6 (2) | 2 (1) |
| Diarrhea | 2 (0.6) | 0 | 40 (19) |
| Anemia^c^ | 2 (0.6) | 0 | 9 (4) |
| Fatigue^c^ | 1 (0.3) | 1 (0.3) | 6 (3) |
| Rash^c^ | 1 (0.3) | 1 (0.3) | 4 (2) |
| ILD^c^ | 1 (0.3) | 0 | 3 (1) |
| Pneumonia^c^ | 1 (0.3)10 | 1 (0.3) | 3 (1) |
| Nausea | 0 | 0 | 12 (6) |
| Upper respiratory tract infection | 0 | 3 (1) | 0 |
| Leukopenia^c^ | 0 | 0 | 5 (2) |
| Hypercreatininemia^c^ | 0 | 0 | 4 (2) |
| Decreased appetite | 0 | 0 | 3 (1) |
| Renal failure | 0 | 0 | 3 (1) |
| **Dose reductions due to TEAEs, n (%)** | **8 (2)** | **0** | **82 (39)** |
| Transaminase elevations^c^ | 2 (0.6) | 0 | 5 (2) |
| Nausea | 1 (0.3) | 0 | 10 (5) |
| Neutropenia^c^ | 1 (0.3) | 0 | 14 (7) |
| Fatigue^c^ | 1 (0.3) | 0 | 8 (4) |
| Diarrhea | 0 | 0 | 38 (18) |
| Rash^c^ | 0 | 0 | 3 (1) |

Discontinuations due to TEAEs in ≥2 patients in any treatment arm and adjustments due to events in ≥3 patients in any treatment arm are shown. Dose adjustments of exemestane were not allowed, while administration of fulvestrant injections later than required per protocol was counted as a dose delay rather than dose interruption.

ALT, alanine aminotransferase; AST, aspartate aminotransferase; ILD, interstitial lung disease; N, number of patients in the total safety population; n, number of patients in the specified category; SOC ET, standard-of-care endocrine therapy; TEAE, treatment-emergent adverse event.

^a^Discontinuations included fatal events due to TEAEs.

^b^One patient in the imlunestrant arm died due to study disease but also had a TEAE of septic shock due to dengue infection that was considered fatal by the investigator

^c^Consolidated terms: Anemia includes anemia and hemoglobin decreased. COVID-19 includes COVID-19, coronavirus infection, and SARS-CoV-2 test positive. Fatigue includes both fatigue and asthenia. Hypercreatinemia includes hypercreatinemia and blood creatine increased. ILD includes interstitial lung disease, pneumonitis, pulmonary fibrosis, and pulmonary toxicity. Leukopenia includes leukopenia and white blood cell count decreased. Neutropenia includes neutropenia and neutrophil count decreased. Pneumonia includes pneumonia and bacterial pneumonia. Rash includes dermatitis acneiform, drug eruption, erythema multiforme, rash pustular, rash, rash erythematous, rash maculo-papular, rash papular, and rash pruritic. Transaminase elevations include increased ALT, increased AST, drug-induced liver injury, increased hepatic enzymes, hepatotoxicity, hypertransaminasemia, and increased transaminases.

**Supplementary Table 3 | Safety in patients <65, 65-74, and ≥75 years of age**

| Relevant TEAEs, n (%) | Imlunestrant  (N=327) | | | Imlunestrant + abemaciclib  (N=208) | | |
| --- | --- | --- | --- | --- | --- | --- |
|  | **<65 years**  **(n=209)** | **65-74 years**  **(n=81)** | **≥75 years**  **(n=37)** | **<65 years**  **(n=118)** | **65-74 years**  **(n=63)** | **≥75 years**  **(n=27)** |
| Patients with ≥1 TEAE | **172 (82)** | **66 (82)** | **32 (86)** | **115 (98)** | **63 (100)** | **26 (96)** |
| Diarrhea | 45 (22) | 21 (26) | 4 (11) | 102 (86) | 55 (87) | 22 (81) |
| Fatigue^a^ | 44 (21) | 13 (16) | 17 (46) | 43 (36) | 25 (40) | 12 (44) |
| Nausea | 34 (16) | 17 (21) | 5 (14) | 50 (42) | 35 (56) | 16 (59) |
| Arthralgia | 30 (14) | 11 (14) | 5 (14) | 11 (9) | 4 (6) | 4 (15) |
| AST increased | 30 (14) | 8 (10) | 3 (8) | 20 (17) | 11 (18) | 3 (11) |
| ALT increased | 25 (12) | 7 (9) | 2 (5) | 14 (12) | 12 (19) | 2 (7) |
| Anemia^a^ | 20 (10) | 9 (11) | 4 (11) | 49 (42) | 28 (44) | 14 (52) |
| Vomiting | 18 (9) | 10 (12) | 1 (3) | 33 (28) | 25 (40) | 7 (26) |
| Abdominal pain^a^ | 18 (9) | 8 (10) | 3 (8) | 27 (23) | 12 (19) | 2 (7) |
| Thrombocytopenia^a^ | 14 (7) | 1 (1) | 3 (8) | 24 (20) | 9 (14) | 5 (19) |
| Leukopenia^a^ | 13 (6) | 4 (5) | 0 | 29 (25) | 18 (29) | 7 (26) |
| Decreased appetite | 13 (6) | 10 (12) | 3 (8) | 23 (20) | 9 (14) | 9 (33) |
| Hypercreatinemia^a^ | 2 (1) | 6 (7) | 1 (3) | 24 (20) | 14 (22) | 7 (26) |
| Neutropenia^a^ | 12 (6) | 4 (5) | 1 (3) | 61 (52) | 29 (46) | 10 (37) |
|  |  |  |  |  |  |  |
| Patients with ≥1 grade ≥3 TEAE | 40 (19) | 12 (15) | 4 (11) | 54 (46) | 32 (51) | 15 (56) |
| Patients with ≥1 SAE^b^ | 24 (12) | 7 (9) | 3 (8) | 19 (16) | 12 (19) | 4 (15) |
| Discontinuations due to AEs^b^ | 9 (4) | 3 (4) | 2 (5) | 5 (4) | 3 (5) | 5 (19) |
| Dose reductions due to AEs | 5 (2) | 3 (4) | 0 | 38 (32) | 27 (43) | 17 (63) |
| Dose interruptions/delays due to AEs | 23 (11) | 9 (11) | 2 (5) | 58 (49) | 40 (64) | 17 (63) |

TEAEs reported in ≥15% of patients in any treatment arm are shown.

AE, adverse event; ALT, alanine aminotransferase; AST, aspartate aminotransferase; N, number of patients in the total safety population; n, number of patients in the specified category; SAE, serious adverse event; TEAE, treatment-emergent adverse event.

^a^Consolidated terms.

^b^Deaths were included as SAEs and discontinuations due to AEs.

**Supplementary Table 4 | Safety in patients harboring tumors with *ESR1* mutations**

| **Relevant TEAEs, n (%)** | **Imlunestrant**  **(N=137)** | | | **SOC ET**  **(N=117)** | | | **Imlunestrant + abemaciclib**  **(N=66)** | |
| --- | --- | --- | --- | --- | --- | --- | --- | --- |
|  | **Any grade** | | **Grade ≥3** | **Any grade** | | **Grade ≥3** | **Any grade** | **Grade ≥3** |
| **Patients with ≥1 TEAE** | **123 (90)** | 26 (19) | | **93 (80)** | 26 (22) | | **65 (99)** | 35 (53) |
| Fatigue^a^ | 35 (26) | | 1 (0.7) | 17 (15) | | 1 (0.9) | 33 (50) | 4 (6) |
| Diarrhea | 33 (24) | | 0 | 15 (13) | | 0 | 58 (88) | 7 (11) |
| Nausea | 33 (24) | | 1 (0.7) | 20 (17) | | 0 | 35 (53) | 1 (2) |
| Arthralgia | 22 (16) | | 0 | 18 (15) | | 0 | 7 (11) | 0 |
| Back pain | 18 (13) | | 1 (0.7) | 4 (3) | | 0 | 3 (5) | 1 (2) |
| Vomiting | 17 (12) | | 1 (0.7) | 7 (6) | | 0 | 23 (35) | 1 (2) |
| Decreased appetite | 16 (12) | | 1 (0.7) | 6 (5) | | 1 (0.9) | 14 (21) | 1 (2) |
| AST increased | 16 (12) | | 0 | 13 (11) | | 1 (0.9) | 8 (12) | 1 (2) |
| Headache | 14 (10) | | 0 | 9 (8) | | 0 | 9 (14) | 0 |
| Constipation | 14 (10) | | 0 | 8 (7) | | 0 | 5 (8) | 0 |
| Cough^a^ | 13 (10) | | 0 | 3 (3) | | 0 | 6 (9) | 0 |
| ALT increased | 12 (9) | | 0 | 10 (9) | | 1 (0.9) | 7 (11) | 2 (3) |
| Anemia^a^ | 12 (9) | | 3 (2) | 16 (14) | | 5 (4) | 26 (39) | 5 (8) |
| Abdominal pain | 11 (8) | | 1 (0.7) | 5 (4) | | 1 (0.9) | 18 (27) | 0 |
| Thrombocytopenia^a^ | 9 (7) | | 3 (2) | 2 (2) | | 2 (2) | 12 (18) | 0 |
| Leukopenia^a^ | 8 (6) | | 1 (0.7) | 1 (0.9) | | 0 | 19 (29) | 3 (5) |
| Neutropenia^a^ | 7 (5) | | 4 (3) | 2 (2) | | 2 (2) | 41 (62) | 21 (32) |
| Hypercreatiaemia^a^ | 3 (2) | | 0 | 3 (3) | | 0 | 15 (23) | 0 |
|  |  | | |  | | |  | |
| Patients with ≥1 SAE^b^ | 16 (12) | | | 18 (15) | | | 11 (17) | |
| Patients who died due to TEAE on study treatment | 2 (1.5) | | | 1 (0.9) | | | 1 (1.5) | |
| Dose reductions | 5 (4) | | | 0 | | | 30 (46) | |
| Dose withheld/omitted | 16 (12) | | | 1 (0.9) | | | 42 (64) | |
| Treatment discontinued due to AE^b^ | 6 (4) | | | 1 (0.9) | | | 4 (6) | |
| Median time on therapy (min-max), days | 169 (11-864) | | | 138 (28-839) | | | 265 (1-695) | |

The most frequent TEAEs reported for ≥10% of patients in any treatment arm are shown.

ALT, alanine transferase; AST, aspartate transferase; N, number of patients in the total safety population; max, maximum; min, minimum; n, number of patients in the specified category; SAE, serious adverse event; SOC ET, standard-of-care endocrine therapy.

^a^Consolidated terms.

^b^Deaths were included as SAEs and discontinuations due to adverse events.

**Supplementary Table 5 | Safety in EMBER-3 and other abemaciclib-containing combinations across trials**

| **TEAEs in <20% of patients, %** | **EMBER-3:**  **Imlunestrant + abemaciclib**  **(N=208)** | | **MONARCH 2^1^:**  **Fulvestrant + abemaciclib**  **(N=441)** | | | **postMONARCH^2^:**  **Fulvestrant + abemaciclib**  **(N=181)** | | |
| --- | --- | --- | --- | --- | --- | --- | --- | --- |
|  | **Any grade** | **Grade ≥3** | **Any grade** | **Grade ≥3** | | **Any grade** | **Grade ≥3** | |
| Diarrhea | 86 | 8 | 86 | 13 | | 75 | 4 | |
| Nausea | 49 | 2 | 45 | 3 | | 33 | 3 | |
| Neutropenia | 48 | 20 | 46 | 24 | | 41 | 25 | |
| Anemia | 44 | 8 | 29 | 7 | | 35 | 11 | |
| Fatigue | 39 | 5 | 40 | 3 | | 33 | 3 | |
| Vomiting | 31 | 1 | 26 | 1 | | 20 | 2 | |
| Leukopenia | 26 | 4 | 28 | 9 | | 18 | 8 | |
| Hypercreatininemia | 22 | 1 | 12 | 1 | | 11 | 0 | |
| Abdominal pain | 20 | 2 | 35 | 3 | | 24 | 2 | |
| Decreased appetite | 20 | 1 | 27 | 1 | | 18 | 1 | |
|  |  |  |  |  | |  |  | |
| Dose reductions due to AEs | 39 | | 43 | | 30 | | |  |
| Dose discontinuations due to AEs | 6 | | 16 | | 6 | | |  |

A total of 137 patients in the imlunestrant + abemaciclib arm from the EMBER-3 safety population were previously treated with a CDK4/6i. None of the 441 patients in the MONARCH 2 trial and all 181 patients in the postMONARCH trial were previously treated with a CDK4/6i.

CDK4/6i, cyclin-dependent kinase 4/6 inhibitor; N, number of patients; TEAE, treatment-emergent adverse event.

**Supplementary Table 6 | Full List of Ethics Committees**

| **Ethics Review Board’s Name** |
| --- |
| LEC: Instituto de Investigaciones Clínicas Mar del Plata  Argentina |
| LEC: Comité Institucional de Ética de la investigación en Salud Fundación CEMAIC  Argentina |
| LEC: FUNDACIÓN CENIT PARA LA INVESTIGACIÓN EN NEUROCIENCIAS  Argentina |
| LEC: CIPREC  Argentina |
| LEC: Clinica Viedma  Argentina |
| LEC: Centro Oncologico Norte  Argentina |
| LEC: Instituto Argentino de Diagnóstico y Tratamiento (IADT)  Argentina |
| LEC: Comité de Ética en Investigación del Ministerio de Salud Publica San Juan  Argentina |
| CLEC: St Vincent’s Hospital Melbourne Human Research Ethics Committee  Australia |
| LEC: Maroondah Hospital  Australia |
| LEC: Eastern Health Human Research Ethics Committee  Australia |
| LEC: St Andrew’s Hospital Ethics Committee  Australia |
| LEC: Abteilung für Gynäkologie / Onkologie  Austria |
| ERB: EK der Medizinischen Universität Wien  Austria |
| LEC: Universitätsklinik für Frauenheilkunde  Austria |
| LEC: Commissie voor medische Ethiek  Belgium |
| LEC: Ethische commissie Onderzoek UZ / KU Leuven  Belgium |
| LEC: Institut Jules Bordet  Belgium |
| LEC: UZB - Commissie Medische ethiek  Belgium |
| LEC: AZ Nikolaas  Belgium |
| LEC: Oncology  Belgium |
| ERB: Clínica de Pesquisa e Centro de Estudos em Ginecologia Oncológica e Mamária LTDA  Brazil |
| LEC: Faculdade de Medicina do ABC  Brazil |
| LEC: IBCC - Instituto Brasileiro de Controle do Cancer  Brazil |
| LEC: Hospital de Cancer de Londrina  Brazil |
| LEC: ICESP - Instituto Do Cancer Do Estado De Sao Paulo  Brazil  Conep - Comissao Nacional De Etica E Pesquisa  Brazil |
| Ethics Committee of The First Affiliated Hospital of Henan University of Science & Technology  China |
| Ethics Committee of Ningbo Medical Center Lihuili Hospital  China |
| LEC: Ethics Committee of Sichuan Cancer Hospital  China |
| LEC: The First Affiliated Hospital of Xi’an Jiaotong University  China |
| LEC: Office of National Drug Clinical Trial Institute, Tianjin Cancer Hospital  China |
| LEC: EC of Hunan Cancer Hospital  China |
| LEC: Ethics Committee for Drug Clinical Trials, Huazhong University of Science and Technology  China |
| LEC: EC of Xiangya Hospital Central South University  China |
| LEC: Ethics Committee of Sun Yat-Sen University Cancer Centre  China |
| LEC: Medical Ethics Committee of Zhejiang Cancer Hospital  China |
| LEC: The First People’s Hospital of Changde Ethics Committee  China |
| LEC: Clinical Research Ethics Committee, Wuhan University People's Hospital  China |
| LEC: Ethics Committee of Jinan Central Hospital  China |
| LEC: EC of the Second Hospital of Jilin University  China |
| Ethics Committee of Ningbo Second Hospital  China |
| ERB: Harbin Medical University Cancer Hospital  China |
| ERB: Jiangsu Provincial People’s Ethics Committee China |
| ERB: Jiangmen Center Hospital Ethics Committee  China |
| LEC: Etická Komise Oblastní Nemocnice Příbram a.s. Onkologie  Czech Republic |
| ERB: CPP Sud-Mediterranee IV  France |
| CIRB: CPP Sud-Mediterranee IV  France |
| CIRB: Ethik-Kommission bei der Landesaerztekammer Hessen  Germany |
| General Hospital of Thessaloniki Papageorgiou  Greece |
| CLEC: Oncology Unit Loc. 1 Euromedica General Clinic of Thessaloniki  Greece |
| CLEC: Oncology Department European Interbalkan Medical Center  Greece |
| CLEC: 2nd Medical Oncology Department, Agios Savvas Regional Cancer Hospital  Greece |
| CLEC: Oncology Clinic, University General Hospital of Larissa  Greece |
| CLEC: Oncology Department, Alexandra Hospital  Greece |
| CLEC: Internal Medicine-Oncology, University General Hospital of Heraklion  Greece |
| LEC: HCG Cancer Centre  India |
| LEC: Lifepoint Multispeciality Hospital  India |
| LEC: Rashtrasant Tukdoji Regional Cancer Hospital  India |
| LEC: HCG-Bangalore  India |
| LEC: IEC, Government Medical College and Hospital  India |
| LEC: Regional Cancer Centre – Thiruvananthapuram  India |
| LEC: HCG Manavata Cancer Centre, Oncology & Hematology  India |
| LEC: Deenanath Mangeshkar Hospital & Research Centre  India |
| LEC: Medstar Speciality Hospital Ethics Committee  India |
| LEC: Yashoda Hospitals  India |
| LEC: Comitato Etico Unico Regionale del Friuli-Venezia-Giulia  Italy |
| LEC: Comitato Etico Regione Liguria  Italy |
| CIRB: Comitato Etico Campania 3  Italy |
| LEC: Nagoya University Hospital  Japan |
| LEC: Kanagawa Cancer Center  Japan |
| LEC: National Hospital Organization Shikoku Cancer Center Institutional Review Board  Japan |
| LEC: Social Medical Corporation Hakuaikai Sagara Hospital  Japan |
| LEC: Chiba Cancer Center  Japan |
| LEC: Niigata Cancer Center Hospital Institutional Review Board  Japan |
| LEC: Hyogo Cancer Center Institutional Review Board  Japan |
| LEC: Kumamoto Shinto General Hospital  Japan |
| LEC: National Hospital Organization Hokkaido Cancer Center Institutional Review Board  Japan |
| LEC: Hiroshima City Hiroshima Citizens Hospital  Japan |
| LEC: Showa University Hospital Institutional Review Board  Japan |
| LEC: University of Tsukuba Hospital Institutional Review Board  Japan |
| LEC: Shizuoka General Hospital  Japan |
| LEC: St. Marianna University Group institutional Review Board  Japan |
| LEC: Gunma Prefectural Cancer Center  Japan |
| LEC: National Hospital Organization Kyushu Cancer Center  Japan |
| LEC: Osaka Prefectural Hospital Osaka International Cancer Institute  Japan |
| CLEC: Sugiura Clinic Institutional Review Board  Japan |
| LEC: Juntendo University Hospital  Japan |
| LEC: Yonsei University Institutional Review Board  Republic of Korea |
| LEC: Samsung Medical Center  Republic of Korea |
| IRB of Kyungpook National University Chilgok Hospital  Republic of Korea |
| LEC: Yeungnam University Medical Center  Republic of Korea |
| LEC: Ajou University Hospital IRB  Republic of Korea |
| LEC: Dong-A University Hospital IRB  Republic of Korea |
| LEC: Kangbuk Samsung Hospital  Republic of Korea |
| LEC: Asan Medical Center IRB  Republic of Korea |
| LEC: Seoul National Univ. College of Medicine, Medical Research IRB  Republic of Korea |
| LEC: Gangnam Severance Hospital IRB  Republic of Korea |
| LEC: Soon Chun Hyang University Hospital Cheonan IRB  Republic of Korea |
| LEC: Gachon University Gil Medical Center IRB  Republic of Korea |
| LEC: Centro Medico Zambrano Hellion  Mexico |
| LEC: Unidad Médica Onco-hematológica  Mexico |
| LEC: Comité de Ética en Investigación de Investigación Farmacológica y Biofarmacéutica S. A. P. I de C. V.  Mexico |
| LEC: Comité de Ética en Investigación de la Unidad de Investigación en Salud de Chihuahua  Mexico |
| LEC: Private Practice - Dr. Joaquin Reinoso  Mexico |
| CLEC: Clinica Bajio CLINBA, S.C.  Mexico |
| LEC: Comité de Ética en Investigación de Oaxaca Site Management Organization S.C.  Mexico |
| LEC: Centro de Investigación Clínica de Alta Especialidad  Mexico |
| LEC: Comite De Etica De Grupo Medico Camino  Mexico |
| ERB: Stichting Beoordeling Ethiek Biomedisch Onderzoek  Netherlands |
| ERB: Stichting Beoordeling Ethiek Biomedisch Onderzoek  Netherlands  COV:  H. Dunantweg 2,  Netherlands |
| LEC: NMRC of Oncology named after N.N.Petrov of MoH of Russia  Russian Federation |
| LEC: Volgograd Regional Clinical Cancer Clinic No.1  Russian Federation |
| ERB: Hospital Universitari Vall d’Hebron  Spain |
| CLEC: CEIm Hospital Universitari Vall d’Hebron  Spain |
| CLEC: Hospital Universitari Vall d’Hebron  Spain |
| LEC: Comité de Ética de la Investigación con Medicamentos (CEIm) provincial de Sevilla y Comité de Ética de la -T  Spain |
| LEC: Chi Mei Hospital - Liouying Branch  Taiwan |
| LEC: National Cheng-Kung Uni. Hospital  Taiwan |
| LEC: Taichung Veterans General Hospital  Taiwan |
| LEC: Koo Foundation Sun Yat-Sen Cancer Center  Taiwan |
| LEC: National Taiwan University Hospital  Taiwan |
| CLEC: Malatya Ethics Committee  Turkey |
| LEC: Central committee, Malatya Clinical Trials Ethics Committee  Turkey |
| CIRB: Malatya Ethics Committee  Turkey |
| LEC: Malatya Ethics Committee  Turkey |
| LEC: Göztepe Professor Dr. Süleyman Yalçın City Hospital  Turkey |
| LEC: Acıbadem Sağlık Kuruluşları Tıbbi Araştırma Etik Kurulu  Turkey |
| LEC: Central City Clinical Hospital of Uzhhorod City Council, therapeutic department  Ukraine |
| LEC: MI Kryvyi Rih Oncology Dispensary of Dnipropetrovsk Regional Council; Chemotherapy Department  Ukraine |
| LEC: Ethics Committee at Municipal Non-Profit Enterprise Odesa Regional Clinical Hospital  Ukraine |
| CLEC: Banner MD Anderson Cancer Center  United States |
| ERB: WCG IRB  United States |
| LEC: Kaiser Permanente Moanalua Medical Center  United States |
| LEC: Avera Central Services IRB #3 – Oncology  United States |
| LEC: WCG IRB  United States |
| ERB: US Oncology Texas Oncology - The Woodlands  United States |
| ERB: US Oncology Texas Oncology Denton  United States |
| ERB: IU Health Ball Memorial Cancer Center  United States |
| ERB: US Oncology Willamette Valley Cancer Institute & Research Ctr.  United States |
| ERB: US Oncology Inc. Institutional Review Board  United States |
| CIRB: WCG IRB  United States |
| ERB: US Oncology Texas Oncology - Denison  United States |
| ERB: US Oncology Texas Oncology - Paris  United States |
| ERB: US Oncology IRB Texas Oncology - Austin Central  United States |
| ERB: US Oncology Texas Oncology - San Antonio Medical Center  United States |
| ERB: US Oncology Texas Oncology - Carrollton  United States |
| CLEC: WIRB-Copernicus Group WCG  United States |
| LEC: Medicine - Section of Hematology/Oncology – Breast  United States |
| LEC: IRB, University of California, Davis  United States |
| ERB: US Oncology Texas Oncology-Baylor Charles A. Sammons Cancer Center  United States |
| ERB: US Oncology Rocky Mountain Cancer Center  United States |
| LEC: The University of Vermont Medical Center Inc  United States |
| ERB: Memorial Sloan Kettering Cancer Center  United States |
| ERB: US Oncology Texas Oncology - Dallas Presbyterian Hospital  United States |

**References**

1. Sledge Jr., G. W. et al. MONARCH 2: Abemaciclib in combination with fulvestrant in women with HR+/HER2- advanced breast cancer who had progressed while receiving endocrine therapy. *J. Clin. Oncol.* **35**, 2875-2884 (2017).
2. Kalinsky, K. et al. Abemaciclib plus fulvestrant in advanced breast cancer after progression on CDK4/6 inhibition: results from the phase III postMONARCH trial. *J. Clin. Oncol.* **43**, 1101-1112 (2025).
